# Analysis of Sociodemographic, Psychological, and Genetic Factors Contributing to Depressive symptoms in Pre-, Peri- and Postmenopausal Women

**DOI:** 10.3390/ijerph15040712

**Published:** 2018-04-10

**Authors:** Elżbieta Grochans, Małgorzata Szkup, Artur Kotwas, Jacek Kopeć, Beata Karakiewicz, Anna Jurczak

**Affiliations:** 1 Department of Nursing, Pomeranian Medical University in Szczecin, ul. Żołnierska 48, 71-210 Szczecin, Poland; grochans@pum.edu.pl (E.G.); szkup.m1@gmail.com (M.S.); 2 Department of Public Health, Pomeranian Medical University in Szczecin, ul. Żołnierska 48, 71-210 Szczecin, Poland; artur.kotwas@pum.edu.pl (A.K.); beata.karakiewicz@pum.edu.pl (B.K.); 3 Faculty of Medicine, School of Population and Public Health, The University of British Columbia, 2206 East Mall Vancouver, BC V6T 1Z3, Canada; jkopec@arthritisresearch.ca; 4 Department of Clinical Nursing, Pomeranian Medical University in Szczecin, ul. Żołnierska 48, 71-210 Szczecin, Poland

**Keywords:** depressive symptoms, premenopause, perimenopause, postmenopause, anxiety, personality

## Abstract

Depressive symptoms that are faced by women in the pre-, peri-, and postmenopausal periods are determined by a wide array of sociodemographic, psychological, and biological variables. The aim of our study was to identify factors that contribute to depressive problems at this stage of life. The study included 815 healthy Polish women aged 45–60 years. The survey part was conducted using the Beck Depression Inventory (BDI), the State–Trait Anxiety Inventory (STAI), the Neuroticism–Extroversion–Openness Five Factor Inventory (NEO-FFI), and a self-developed questionnaire. Genetic analysis was also performed. Depressive symptoms were observed in 25.5% of participants. 70% of the women were postmenopausal. No statistically significant differences in the severity of depressive symptoms were demonstrated with regard to genetic variables (*p* > 0.05). Reproductive capacity (*p* < 0.001), employment (*p* < 0.001), and being married (*p* < 0.018) were found to reduce the incidence of depressive symptoms. The contribution of personality and anxiety as a trait to depressive symptoms varied. Conclusions: The factors predisposing pre-, peri-, and postmenopausal women to depressive symptoms include lower education, lack of a life partner, unemployment, high anxiety, and neurotic personality. No evidence was found for the contribution of genetic factors to depressive symptoms in the examined women.

## 1. Introduction

Natural menopause is a quantitative biological feature that indicates the end of a woman’s reproductive capacity in the physiological process of aging [1]. According to the World Health Organization’s 1996 definition, menopause is the final normal menstruation in a woman’s life, resulting from a complete loss of ovarian follicles, followed by a break of at least one year in menstrual bleeding [2].

As suggested by the WHO, a woman’s menopausal life can be divided into three successive stages: premenopause, defined as the whole of the reproductive period prior to menopause, or as the one or two years immediately before menopause, which are associated with irregularity in menstrual bleeding; this usually takes place at about 45–49 years of age [3,4]; perimenopause is the period immediately prior to menopause (when the endocrinological, biological, and clinical features of approaching menopause commence) and the first year after menopause; postmenopause dates from the final menstrual period, regardless of whether the menopause was induced or was spontaneous [5].

### 1.1. Perimenopausal Period

Depression affects approximately 350 million people, and the probability of being affected by it is rising throughout the world [6]. The risk of depression and of severe depressive symptoms is higher during the menopausal transition and early postmenopause than in the premenopausal period [7,8,9,10]. A special role in this process is attributed to estrogens, which modulate women’s mood and whose deficiency can result in depressive symptoms. It is estimated that 8–40% of perimenopausal women suffer from depressive disorders [11]. The connection between depression and the perimenopausal period has been explored in many studies, which have, however, provided inconsistent results [12,13,14]. The incidence of depression in this population may depend on a wide range of sociodemographic, psychological, and biological factors. In our study, we therefore attempted to establish the link between selected factors and depressive symptoms in perimenopausal and postmenopausal women.

### 1.2. Sociodemographic Factors

Depression and other psychiatric disorders may be underlain by sociodemographic factors, such as age, sex, marital status, education, and income. Nonetheless, the nature of this connection is unclear and debated [15].

One of the essential protective factors is a third-level education, whose importance has been demonstrated in many studies—including multinational and multiethnic studies [11,16,17,18]. The available studies show that a higher level of education minimizes unpleasant menopause-related symptoms, whereas lower education is accompanied by a greater tendency to swinging or unstable moods [8].

The place of permanent residence can contribute to the incidence of depression. Some studies suggest that women living in cities are less prone to depression than those in rural areas [19,20].

A regular life partner is not always regarded as a protective factor against depressive symptoms. In the study of 45–55-year-old women, Jagtap et al. did not observe such an association [11].

The significance of work to the emotional well-being of perimenopausal women is debatable—while in a study of women from India, no such relationship was observed [11,21], a study of Brazilian women provided evidence that an active working life protects against depression [22].

### 1.3. Genetic Background

Genetic factors can enhance the risk of depressive symptoms [23]. One of the genes that may be responsible for psychiatric problems is *MAOA,* which is located on the short arm of the X chromosome. This locus affects the activity of functional polymorphisms depending on sex. Polymorphisms in the human *MAOA* gene have a variable number of tandem repeats (VNTR), which has effects on the *MAOA* transcriptional activity [24]. Due to their function—which mainly involves the regulation of the serotonin, dopamine, and norepinephrine levels—polymorphic forms of the *MAOA* gene are regarded as important contributors to individual differences in psychological characteristics and the severity of psychiatric disorders [23,25]. Some reports indicate that the *MAOA* gene is related to depressive symptoms and major depressive disorders [26,27].

Research on the relationship between 5-HTT and pathophysiology of depression started as early as in the eighties of the twentieth century [28]. It has been noticed that in a normally functioning brain, after the release to the synaptic cleft, serotonin is taken up, recycled, and released again by presynaptic neurons. The goal of this process is to eliminate excessive serotonergic activity. Excessive 5-HTT activity can result in abnormally low extracellular serotonin levels, and so contribute to mood disorders and other psychiatric problems, including depression [29]. One of the most commonly analyzed genes having an impact on serotonergic pathways is *SLC 6A4*, encoding 5-HTT. The 5-HTT gene polymorphism emerges as a result of the insertion or deletion of a 44-bp fragment, which determines its transcriptional activity [30]. The short (s) allele entails a threefold decrease in transcriptional activity as compared to the long (l) allele. This, in turn, can increase the risk of developing mood disorders, anxiety, and neuroticism in the group of carriers of the (s) variant of this gene polymorphism [31].

### 1.4. Selected Psychological Factors

The severity of menopausal symptoms can also be determined by personality factors. Numerous studies suggest that characteristics, such as optimism, self-confidence, and aggression are associated with means of reacting to and coping with menopausal symptoms, whether psychological (mood disorders, depression), psychosomatic, or vasomotor [32]. An important personality trait contributing to menopause-related problems (including depression) is neuroticism. Higher levels of this trait involve a general tendency to experience negative emotions, such as sadness, anxiety, and a sense of guilt, anger, and fear [33]. What is more, highly neurotic women experience more severe physical climacteric symptoms [34]. Neuroticism substantially strengthens psychological reactions to external and internal stress factors [35], thus resulting in more negative perceptions of the changes that typically occur in the menopausal period, and increasing the risk of depressive disorders.

Perimenopausal women with depression often report anxiety symptoms. Anxiety and depressive symptoms often exist together, and the distinction between them is not always clear. Both depressive and anxiety disorders are strongly related to four overlapping problems, namely: difficulty sleeping, concentration problems, fatigue, and psychomotor excitement. A history of anxiety disorders increases the risk of depression at any age, especially among women [36].

Middle-age women often complain of anxiety symptoms. Studies show that as many as 51% of them report tension, irritability, and nervousness lasting for over two weeks preceding the study [37], and 25% of women aged 40–55 years claim that they frequently experience such symptoms [18]. According to some authors, these complaints worsen in the perimenopausal period, and the risk of their development is then significantly higher than before menopause [18,38]. Nevertheless, there are researchers who have not confirmed this relationship [39].

The aim of the study was to seek out and assess sociodemographic, psychological, and genetic factors that contribute to the depressive symptoms faced by perimenopausal and postmenopausal women.

## 2. Materials and Methods

The study was conducted among 815 healthy women aged 45–60 years from north-west Poland. West Pomeranian voivodeship is the fifth-largest province of Poland, covering 7.3% of the country’s area. The population density is 75 people per 1 km^2^. City dwellers constitute 68.5% of the population of this province. There are two cities with populations of over 100,000, and the rest of the area includes 22 cities of up to 100,000 residents and of up to 10,000 residents, as well as numerous villages.

The participants were recruited based on information posters in public places and advertisements in the local press. The criteria for inclusion in the study were: 45–60 years of age, menopausal status confirmed by an interview and hormone testing (the levels of follicle-stimulating hormone (FSH), luteinizing hormone (LH), and anti-Müllerian hormone (AMH)), intentional written consent to take part in the study, and no history of cancerous, metabolic (diabetes, thyroid diseases), gynecological, or mental diseases.

Women were excluded if they did not meet the above criteria. ICD-10 psychiatric disorders were excluded in all of the participants by means of the Primary Care Evaluation of Mental Disorders (PRIME-MD) questionnaire, a screening tool for clinical psychiatric disorders.

All of the subjects gave their informed consent for inclusion in the study. The study was conducted in accordance with the Declaration of Helsinki, and the protocol was approved by the Bioethical Commission of the Pomeranian Medical University in Szczecin (permission numbers: KB-0080/187/09, KB-0012/104/11, KB-0012/12/12)*.*

The data for this study were collected as part of a larger investigation.

### 2.1. Research Procedure

The study involved diagnostic survey and genetic (laboratory) analysis. The following standardized research instruments were applied:

1. The Beck Depression Inventory (BDI) for the assessment of depressive symptoms [40]. This is a self-administered, 21-question instrument. Participants rate items on a four-point Likert scale from 0 (the least severe symptoms) to 3 (the most severe symptoms).

The total number of points reflects the severity of depressive symptoms. We assumed the following score ranges: depressive symptoms—scores of 20 points and more; no depressive symptoms—scores below 20.

2. State-Trait Anxiety Inventory (STAI) for measuring the level of anxiety. This consists of two independent parts, each including a set of 20 questions. The first part, STAI (X-1), measures the level of anxiety understood as the transitory and situationally conditioned status of an individual. The second part, STAI (X-2), concerns anxiety understood as a relatively permanent personality trait [25].

Respondents take a stance on each statement, choosing one of four possible answers. The level of anxiety is expressed as the number of points that is obtained through summing up scores for separate answers. Scores for each part of the questionnaire may range from 20 to 80 points. Raw data are converted into standardized results for gender and age (stens). In this study, the authors used the 10-unit sten scale, interpreted as follows:

Scores of 1–4—low results (reflect low levels of anxiety as a trait and as a state)—no anxiety;
Scores of 5–6—average results (reflect an average level of anxiety as a trait and a state)—anxiety within normal ranges;
Scores of 7–10—high results (reflect a high level of anxiety as a trait and a state)—anxiety.

A high score for anxiety as a state may suggest stress associated with a difficult life situation, while a high score for anxiety as a trait indicates a permanent predisposition of a person to react with anxiety to life situations [25].

3 The Neuroticism-Extroversion-Openness-Five Factor Inventory (NEO-FFI), consists of five subscales measuring neuroticism, extroversion, openness to experience, agreeableness, and conscientiousness [41,42]. Sixty self-descriptive statements (12 for each subscale) are rated on a five-point Likert scale, with 1 denoting ‘I absolutely disagree’, 2—‘I disagree’, 3—‘I don’t know’, 4—‘I agree’, and 5—‘I absolutely agree’. The points obtained for particular statements within each subscale are summed up, thus giving scores ranging from 12 to 60. Raw results are then converted into sten scores reflecting the levels of particular traits: high (7–10), average (4–6), or low (1–3) for each of the five scales.

The Cronbach’s alpha for particular NEO-FFI subscales is as follows: neuroticism (0.86), extroversion (0.77), openness to experience (0.73), agreeableness (0.68), and conscientiousness (0.81).

Neuroticism: a high score refers to a sensitive, emotional person with a tendency to worry and feel tense; an average score refers to a person who is generally calm and even-tempered, but sometimes experiences anger, sorrow, and feelings of guilt; a low score describes a calm, relaxed person who endures hardship and copes well with difficult situations.

Extroversion: a high score refers to a sociable, active, and optimistic person; an average score to a moderately sociable and active person who likes contact with others but also values his or her privacy and intimacy; a low score describes a person who treats others with reserve and prefers to be alone or in the company of a few friends.

Openness to experience: a high score describes an imaginative person who is open to new experiences and has a wide array of interests; an average score refers to a person who is practical by nature, but has unconventional interests, and tries to keep a balance between the attachment to the old and the fascination with the new; a low score refers to a practical person, a traditionalist who has his or her feet firmly fixed on the ground.

Agreeableness: a high score refers to a pleasant, non-combative, good-hearted person, sensitive to the problems of others; an average score to a person who is generally nice and friendly, but who can sometimes be competitive; a low score describes a skeptical, competitive person who makes no attempt to conceal his or her dissatisfaction.

Conscientiousness: a high score refers to a person who is well-organized, meticulous, reliable, and persistent, and has strong willpower; an average score to a moderately well-organized person who has clearly defined life goals, though he or she does not aspire to reach them at any cost; a low score describes a spontaneous person who lives for the moment and does not plan anything, is not well-organized, and who values comfort and lounging about.

4 The self-developed questionnaire included questions concerning sociodemographic data (age, education, marital status, place of residence, employment), the dates of the first and the last menstruation, the use of Menopausal Hormone Therapy (MHT) in the case of postmenopausal women, current smear test, mammogram, and ultrasound results, as well as past and current diseases (cancerous, metabolic, mental), taking medicines on a regular basis (for what reasons), and taking supplements.

The second stage of the study involved laboratory analysis. Upon obtaining the women’s assent, venous blood was collected using BD Vacutainer Venous Blood Collection Tubes. The blood was drawn in the treatment room and was delivered to the laboratory in accordance with binding rules and procedures. The FSH ranges accepted in the study as normal were FSH follicular levels—that is, 3.5–12.5 mlU/mL. The levels of FSH and AMH were determined in a laboratory accredited with ISO 9001:2008 quality certification. Genetic testing was based on isolating DNA from whole blood by Miller’s salting-out method [43]. Polymerase chain reaction (PCR) was used to identify DNA polymorphisms. The aim of the analysis was to amplify the fragment consisting of 2–5 repetitions of the 30-bp VNTR polymorphism in the *MAOA* promoter region. The following primer sequences were used: *MAOA* F, 5′ CCC AGG CTG CTC CAG AAA 3′; and, *MAOA* R, 5′ GGA CCT GGG TTG TGC 3′. The sizes of the amplified fragments were as follows: 239, 209, 226, and 269 bp. In the analysis of the 5-HTT gene polymorphism, the fragment including the 44-bp ins/del in the regulatory sequence (the presence or the lack of 44-bp) was amplified. The following primer sequences were used: HTT F, 5′ GGC GTT GCC GCT CTG AAT GC 3′; and, HTT R, GAG GGA CTG AGC TGG ACA ACC AC 3′. The sizes of the amplified fragments were 484 and 528 bp.

### 2.2. Statistical Analysis

Statistical analysis was performed using Statistica 7.1 PL (StatSoft, Kraków, Poland). The distribution type was determined for all of the variables. The Shapiro–Wilk test was employed to verify the normality of the distribution. The chi-squared independence test was used to verify the null hypothesis regarding the independence of variables. The strength of relationships between variables was assessed by Pearson’s linear correlation coefficient and Spearman’s rank–correlation coefficient. We additionally used multiple and simple logistic regression to determine the effect of the explanatory variables on the odds ratio (OR) of a higher risk of depressive symptoms (the BDI), with a 95% confidence interval. The significance level was set to α = 0.05. The power calculated for all of the genetic tests exceeded 0.95 (*p* > 0.95).

## 3. Results

The mean age of the women was 52.6 ± 6.9 and the median was 54.46% of the participants had completed third-level education, and 43.7% had completed secondary education. 72.8% had life partners, 66.9% lived in cities with a population of over 100,000, and 71% were active in the labor market.

25.5% of the women suffered from depressive symptoms, according to the BDI, and 74.5% had no such symptoms. Anxiety as a state was observed in 40.6% of the participants, and anxiety as a trait in 16%. Analysis of personality structure according to the NEO-FFI demonstrated that most of the women had average levels of the following personality traits: neuroticism—44.2%, extroversion—51.4%, agreeableness—57.9%, and conscientiousness—58.9%. The only dimension in which high levels were noted was openness to experience—50.4%.

The incidence of depressive symptoms was analyzed with regard to sociodemographic data. There were statistically significant differences between the women, depending on education—the majority of those with third-level education had no depressive symptoms (*p* < 0.001). Marital status had a substantial impact on the occurrence of depressive symptoms (*p* < 0.018)—depressive symptoms were visibly less common among women having life partners (marriage, cohabitation). Employment was a statistically significant determinant of depressive symptoms (*p* < 0.001), which were definitely more common among women having no active working life. Only place of residence was not a statistically significant contributor to depressive symptoms in women aged 45–60 years (Table 1).

Depressive symptoms were also assessed with regard to the reproductive stage. The participants were divided into menopausal (572; 70%) and still menstruating women (243; 30%). Analysis revealed statistically significant differences (*p* < 0.001)—depressive symptoms were observed in 29% of the postmenopausal women and only 16% of the menstruating ones.

No statistically significant differences in the severity of depressive symptoms according to the BDI were demonstrated in peri- and postmenopausal women, depending on genetic variables (the distribution of the genotypes and alleles of the 44-bp VNTR polymorphism in the 5-HTT (*SLC6A4*) promoter region, and the 30-bp VNTR polymorphism in the *MAOA* promoter region) (*p* > 0.05) (Table 2).

Statistically significant differences in the incidence and severity of depressive symptoms were observed with regard to psychological variables (*p* < 0.001). Both anxiety as a state and anxiety as a trait were considerably more common among women with severe depressive symptoms. Analysis of the relationship between depressive symptoms and personality structure, according to the NEO-FFI, revealed statistically significant differences in the levels of neuroticism, extroversion, agreeableness, and conscientiousness (*p* < 0.001). Higher levels of neuroticism were noticeably more often observed in the women with severe depressive symptoms, whereas high extroversion, agreeableness, and conscientiousness were more common among those without depressive symptoms (Table 3).

Depressive symptoms statistically significantly correlated with age, education, anxiety as a state and as a trait, and all of the personality traits. The correlation with age and education was not strong. Higher anxiety as a state and higher anxiety as a trait entailed more severe depressive symptoms (*r* = 0.345 and *r* = 551, respectively). There was a positive strong correlation between depressive symptoms and neuroticism (*r* = 0.622), and a negative correlation between depressive symptoms and other personality traits (Table 4).

We used multiple logistic regression to assess the effect of selected variables on the incidence of depressive symptoms, according to the BDI. Significant variables associated with depressive symptoms in multiple logistic regression were: employment (OR = 0.368; 95% CI = 0.219 to 0.618), having a life-partner (OR = 0.648; 95% CI = 0.416 to 1.008), anxiety as a trait (OR = 1.640; 95% CI = 1.355 to 1.985), and higher neuroticism (OR = 1.792; 95% CI = 1.560 to 2.059). There was a moderate coincidence between the real data and the model that is presented in our study (R^2^ = 0.493) (Table 5).

## 4. Discussion

### 4.1. Perimenopausal Period

The menopausal transition is a natural stage in a woman’s life, which in most cases proceeds without major difficulties. Nevertheless, quite a number of women experience problems that are associated with physical functioning in this period, as well as anxiety and mood disorders, including depression [6]. Numerous studies show that anxiety is diagnosed in approximately 24.11% and depression in 13.18% of menopausal women [6], however there are differences in the epidemiology of these phenomena between various populations of the world. The study conducted in southern China demonstrated that symptoms of depression affected 21.1% of the participants, and 29.7% complained of pathological anxiety. In Beijing, China, the incidence of depressive symptoms among middle-age women was 24%, in Turkey—25%, in Taiwan—31% to 38.7%, and in Spain—40%. The highest incidence rate for depressive symptoms was recorded in Philadelphia, USA—50% and Mexico—52% [44,45,46,47]. In our study of 45–60-year-old women, depressive symptoms of average and high severity according to the BDI were observed in 25.5% of the participants. Anxiety as a state that was diagnosed on the basis of the STAI was found in 40.6% of the participants, and anxiety as a trait in 16%.

### 4.2. Sociodemographic Factors

Bromberger et al. provided evidence that higher levels of education—associated with higher social support, better prospects for the future, less bothersome menopausal symptoms, and more positive attitudes towards this period of life—act as a buffer against climacteric symptoms. Lower education levels, on the other hand, are more often accompanied by changing moods [18]. The relationship between the level of education (including the ability to read and write) and the severity of depression was analyzed in senior citizens in the USA. It was established that one standard deviation difference in educational attainment, corresponding with approximately a three-year education period, was associated with a 0.35-point decrement in the Centers for Epidemiologic Studies-Depression Scale. These findings, indicating the benefits of education [48], are parallel to those that were obtained in our study. We noticed that higher levels of education considerably diminished the incidence of depressive symptoms—better educated women were at half lower risk of depressive symptoms than their counterparts with primary and secondary education. Different results were obtained by Akhtar-Danesh and Landeen, who observed the smallest proportion of people with depression among those having education lower than secondary [15].

For Arabic menopausal women, their place of residence was a factor contributing to the severity of depression, anxiety, and stress [49]. Some authors also reported on higher incidence of depression among women living in rural areas compared with female city dwellers. This was probably associated with the presence of other protective factors, such as a higher level of women’s education, better financial standing, more social contacts, a higher percentage of employed women, and greater satisfaction with sexual life [19,20]. In the study presented here, a significant difference between women from big cities and those from smaller places was not confirmed.

The meta-analysis of studies concerning stroke patients from sub-Saharan Africa demonstrated that depression was linked to divorced marital status and low levels of education [50]. The contribution of marital status to depressive symptoms was also confirmed among African Americans [51]. The results of our study support these findings—married individuals and those that were living in cohabitation were at a lower risk of depression than single ones.

Many researchers emphasize that people’s functioning on the labor market is related to their physical and mental state. Employment improves health indicators [52], which refers both to mental status [53,54] and general health [55,56]. Warr et al. noticed that, even though some aspects of employment can involve potential health hazards, ceasing to work usually has negative effects on health [57]. Our study substantiated a significant relationship between employment and the incidence of depressive symptoms—gainful employment was a factor reducing the risk of developing depressive disorders.

### 4.3. Genetic Background

The relationship between psychological functioning and genetic background has been the subject of numerous scientific investigations. It was observed that the ‘s’ allele of the 5-HTT gene polymorphism can cause greater impulsiveness, and thus a higher risk of suicide attempts among its carriers [58]. This relationship is especially noticeable if genetic predisposition is enhanced by environmental determinants [59]. In such cases, the manifestation of severe depression can be definitely stronger than in carriers of the ‘l’ allele [60]. Similarly, the presence of the high-activity genotype of the *MAOA* polymorphism, when combined with difficult life events can increase impulsiveness, neuroticism, and aggression [61,62,63]. Our results do not justify the conclusion that depressive symptoms are directly related to particular genotypes and alleles of the 5-HTT and MAO-A gene polymorphisms in perimenopausal women. It is worth emphasizing that the study sample comprised of women coming from general population. Since one of the criteria for inclusion to the study was the lack of mental diseases, none of the participants had a previous diagnosis of clinical depression.

### 4.4. Psychological Factors

In the study conducted by Bal et al., the women that were characterized by high levels of neuroticism had 9.3 times lower quality of life in the psychological functioning domain [64]. Predisposition to depressive disorders in individuals with average and high levels of neuroticism was even more apparent in our study—such women were at a 43 times higher risk of depressive symptoms than those with low levels of this personality trait. Sutin et al., who analyzed people with evidence of cognitive impairment at the end of life, established that the respondents with higher neuroticism were at a significantly greater risk of impaired functioning in many areas, and more often suffered from depression. This association was true, even after exclusion of such interfering factors as age, sex, race, and education [65]. What is more, the study of Taiwanese climacteric women revealed that neuroticism is an important contributor to the persistence of depression [66]. High extroversion, on the other hand, entails better quality of life, resulting, among others, from greater interpersonal support [64]. These observations find confirmation in our study—individuals with higher levels of extroversion, agreeableness, and conscientiousness experience depressive disorders substantially less frequently than those with high neuroticism.

Bromberger et al. provided evidence that middle-aged women with low levels of anxiety prior to menopause showed a tendency towards high anxiety during and after menopause [67]. Previous reports had demonstrated that anxiety disorders increase the risk of later depressive problems, especially among women, and that anxiety symptoms correlate with longer duration of depression, worse response to treatment, and a longer road to recovery [68,69]. Anxiety symptoms are important predictors of depressive disorders, and their monitoring may be a useful clinical instrument, especially with middle-age women [36]. The research carried out among Arabic menopausal and postmenopausal women shows that stress, anxiety, and depression are related to numerous aspects of menopausal symptoms and psychosocial problems [49]. In the study that is described in this article, depressive symptoms were evidently linked to anxiety. We noticed that the risk of developing depressive symptoms was over 2.5 times higher for individuals with the levels of anxiety as a state being above normal ranges, and nearly seven times higher for those with elevated levels of anxiety as a trait.

## 5. Limitations

Our study is limited in several ways. One of the limitations is the fact that we analyzed few women from rural areas and towns up to 10,000 residents. This resulted mainly from the arrangement of the cities in West Pomeranian voivodeship. Delivery of the research material from distant regions to the certified laboratory, where all analyses were performed, was in many cases impossible. We also did not exclude from the study the women who had experienced traumatic life events, such as the death of a close person, a loss of property, a loss of job etc. We assumed that such events may happen throughout the whole life, and in all age brackets, but it is true that using this exclusion criterion would allow us to discriminate between depressive symptoms due to life events and those associated with changes in hormone levels. A final limitation is that information about lack of menstruations for at least one year was only obtained by an interview and was not confirmed by hormone analysis. This is because the study presented here is a part of a larger investigation, and the part concerning postmenopausal women did not require verification of the last menstruation by hormone testing.

## 6. Conclusions

The factors predisposing pre-, peri-, and postmenopausal women to depressive symptoms include lower education, lack of a life partner, unemployment, high anxiety, and neurotic personality. No evidence was found for the contribution of genetic factors to depressive symptoms in the examined women.

## Figures and Tables

**Table 1:** Influence of sociodemographic variables on the incidence of depressive symptoms in peri, pre-, and postmenopausal women.

Variable		The BDI			Chi^2/*p*
		No Depressive Symptoms	Depressive Symptoms	Total	
		*n* (%) 608 (74.6)	*n* (%) 207 (25.4)	*n* (%) 815 (100)	
**Education**	primary	8 (1.3)	8 (3.8)	16 (2.0)	19.783/0.001
	vocational	42 (6.9)	25 (12.1)	67 (8.2)	
	secondary	253 (41.8)	102 (49.3)	355 (43.7)	
	third-level	303 (50.0)	72 (34.8)	375 (46.1)	
**Place of Residence**	rural area	60 (10.1)	19 (9.4)	79 (9.9)	2.537/0.469
	up to 10 000	26 (4.4)	14 (6.9)	40 (5.0)	
	up to 100 000	106 (17.8)	39 (19.3)	145 (18.1)	
	over 100 000	405 (67.7)	130 (64.4)	535 (67.0)	
**Marital Status**	marriage/cohabitation	454 (74.7)	137 (66.2)	591 (72.5)	6.990/0.018
	single	154 (25.3)	70 (33.8)	224 (27.5)	
**Employment**	employed	457 (75.5)	117 (57.1)	574 (70.9)	25.283/0.001
	unemployed	148 (24.5)	88 (42.9)	236 (29.1)	

BDI—the Beck Depression Inventory; *n*—the number of participants.

**Table 2:** The genotype distribution and the frequency of the alleles of the 44-bp variable number of tandem repeats (VNTR) polymorphism in the 5-HTT (*SLC6A4*) promoter region, and the 30-bp VNTR polymorphism in the MAO-A promoter region in the peri, pre- and postmenopausal women.

Variable		The BDI			Chi^2/*p*
		No Depressive Symptoms	Depressive Symptoms	Total	
		*n* (%) 608 (74.6)	*n* (%) 207 (25.4)	*n* (%) 815 (100)	
**Genotype**					
**5-HTT gene**	l/l	250 (41.1)	96 (46.4)	346 (42.5)	1.760/0.415
	l/s	260 (42.8)	80 (38.6)	340 (41.7)	
	s/s	98 (16.1)	31 (15.0)	129 (15.8)	
**MAO-A gene**	3/3	77 (12.7)	21 (10.1)	98 (12.0)	1.394/0.498
	3/4	273 (44.9)	101 (48.8)	374 (45.9)	
	4/4	258 (42.4)	85 (41.1)	343 (42.1)	
**Allele**					
**5-HTT gene**	l	760 (46.6)	272 (16.7)	1032 (63.3)	1.362/0.243
	s	456 (28.0)	142 (8.7)	598 (36.7)	
**MAO-A gene**	3	427 (26.2)	143 (8.8)	570 (35.0)	0.045/0.833
	4	789 (48.4)	271 (16.6)	1060 (65.0)	

BDI—the Beck Depression Inventory; *n*—the number of participants.

**Table 3:** Influence of anxiety and personality on depressive symptoms in the pre-, peri-, and postmenopausal women.

Variable		The BDI			
		No Depressive Symptoms	Depressive Symptoms	Total	Chi^2/*p*
		*n* (%) 608 (74.6)	*n* (%) 207 (25.4)	*n* (%) 815 (100)	
**Anxiety according to the STAI**					
**STAI X-1**	no anxiety	56 (9.2)	1 (0.5)	57 (7.0)	45.362/0.001
	anxiety within normal ranges	342 (56.3)	85 (41.1)	427 (52.4)	
	anxiety	210 (34.5)	121 (58.4)	331(40.6)	
**STAI X-2**	no anxiety	126 (20.7)	2 (1.0)	128 (15.7)	126.485/0.001
	anxiety within normal ranges	431 (70.9)	126 (60.9)	557(68.3)	
	anxiety	51 (8.4)	79 (38.2)	130 (16.0)	
**Personality according to the NEO-FFI**					
**Neuroticism**	low	278 (45.7)	13 (6.3)	291 (35.7)	218.420/0.001
	medium	276 (45.4)	84 (40.6)	360 (44.2)	
	high	54 (8.9)	110 (53.1)	164 (20.1)	
**Extroversion**	low	50 (8.2)	59 (28.5)	109 (13.4)	60.182/0.001
	medium	318 (52.3)	101 (48.8)	419 (51.4)	
	high	240 (39.5)	47 (22.7)	287 (35.2)	
**Openness to experience**	low	61 (10.0)	20 (9.7)	81 (9.9)	2.237/0.327
	medium	232 (38.2)	91 (44.0)	323 (39.6)	
	high	315 (51.8)	96 (46.3)	411 (50.5)	
**Agreeableness**	low	57 (9.4)	46 (22.2)	103 (12.7)	36.714/0.001
	medium	345 (56.7)	127 (61.4)	472 (57.9)	
	high	206 (33.9)	34 (16.4)	240 (29.4)	
**Conscientiousness**	low	51 (8.4)	52 (25.1)	103 (12.6)	49.501/0.001
	medium	358 (58.9)	122 (59)	480 (58.9)	
	high	199 (32.7)	33 (15.9)	232 (28.5)	

BDI—the Beck Depression Inventory; STAI-X1—the State Anxiety Inventory; STAI-X2—the Trait Anxiety Inventory; NEO-FFI—the Neuroticism-Extroversion-Openness-Five Factor Inventory; *n*—the number of participants.

**Table 4:** Pearson’s linear correlations between the Beck Depression Inventory (BDI) results and selected variables.

Variable	The BDI	
	*r*	*p*
Age (*n* = 813)	0.15	0.001
Education (*n* = 813)	−0.223	0.001
Place of residence (*n* = 799)	−0.026	0.466
Anxiety as a state according to the STAI X-1 (sten scores) (*n* = 811)	0.345	0.001
Anxiety as a trait according to the STAI X-2 (sten scores) (*n* = 810)	0.551	0.001
Personality according to the NEO-FFI—neuroticism (sten scores) (*n* = 815)	0.622	0.001
Personality according to the NEO-FFI—extroversion (sten scores) (*n* = 815)	−0.294	0.001
Personality according to the NEO-FFI—openness to experience (sten scores) (*n* = 815)	−0.108	0.002
Personality according to the NEO-FFI—agreeableness (sten scores) (*n* = 815)	−0.249	0.001
Personality according to the NEO-FFI—conscientiousness (sten scores) (*n* = 815)	−0.245	0.001

BDI—the Beck Depression Inventory; STAI-X1—the State Anxiety Inventory; STAI-X2—the Trait Anxiety Inventory; NEO-FFI—the Neuroticism-Extroversion-Openness-Five Factor Inventory; *n* —the number of participants; *r*—Pearson’s correlation coefficient; *p*—statistical significance.

**Table 5:** Multiple logistic regression analysis of the influence of selected variables on depressive symptoms according to the BDI.

Variable	*p*	OR	95% CI
Age	0.928	0.998	0.948–1.050
Reproductive capacity	0.190	0.600	0.280–1.287
Employment	0.001	0.368	0.219–0.618
Marital status	0.054	0.648	0.416–1.008
Education	0.666	1.106	0.701–1.743
STAI X2	0.001	1.640	1.355–1.985
NEO-FFI neuroticism	0.001	1.792	1.560–2.059
NEO-FFI openness to experience	0.020	1.148	1.022–1.290
NEO-FFI extroversion	0.237	0.932	0.828–1.048
NEO-FFI agreeableness	0.097	0.889	0.774–1.021
NEO-FFI conscientiousness	0.001	0.789	0.697–0.893
Constant variable	0.017	0.013	-

*p*—statistical significance; OR—odds ratio; CI—confidence interval; STAI-X2—the Trait Anxiety Inventory; NEO-FFI—the Neuroticism-Extroversion-Openness-Five Factor Inventory.
